# One Health Investigation into Fatal Encephalitis Caused by Pigeon Paramyxovirus Type 1, France

**DOI:** 10.3201/eid3205.251576

**Published:** 2026-05

**Authors:** Nicolas Veyrenche, Susana Boluda, Philippe Pérot, Isabelle Malissin, Marianne Leruez-Ville, Anne Jamet, Agnès Ferroni, Béatrice Regnault, Maud Salmona, Linda Feghoul, Laurine Robert-Capraro, Aurélie Leroux, Béatrice Grasland, Eric Niqueux, François-Xavier Briand, Isabelle Plu, Danielle Seilhean, Bruno Megarbane, Jacques Fourgeaud, Nolwenn M. Dheilly

**Affiliations:** Hôpital Necker-Enfants malades, Paris, France (N. Veyrenche, M. Leruez-Ville, A. Jamet, A. Ferroni, J. Fourgeaud); Université Paris Cité, FETUS, Paris (N. Veyrenche, M. Leruez-Ville, J. Fourgeaud); APHP-Hôpital de la Pitié-Salpêtrière, Sorbonne Université, Paris (S. Boluda, I. Plu, D. Seilhean); Institut Pasteur, Université de Paris, Paris (P. Pérot, B. Regnault, L. Robert-Capraro, N.M. Dheilly); Lariboisière Hospital, Paris Cité University, INSERM UMRS-1144, Paris (I. Malissin, L. Feghoul, B. Megarbane); Saint-Louis Hospital, APHP, Paris (M. Salmona); Biology and Pathogenesis of Viral Infection team, INSERM UMR 1342, Saint Louis Research Institute, Université Paris-Cité, Paris (M. Salmona); Anses, Ploufragan-Plouzané-Niort Laboratory, Ploufragan, France (A. Leroux, B. Grasland, E. Niqueux, F.-X. Briand)

**Keywords:** Pigeon Paramyxovirus type 1, metagenomics, meningitis/encephalitis, One Health, phylogeny, neuropathology, zoonoses, viruses, France

## Abstract

Pigeon paramyxovirus type 1 (PPMV-1) is a genotype of avian paramyxovirus type 1 that uses species of the family Columbidae as reservoir species. We report fatal PPMV-1 encephalitis in a human without immunosuppression or travel history outside metropolitan France. Postmortem analyses revealed PPMV-1 in tissues, underscoring that physicians should consider this potential diagnosis.

Paramyxoviridae is a family of enveloped, negative-sense single-stranded RNA viruses that includes many human pathogens. Paramyxoviruses have a broad host range and high risk for spillover events to humans (*1*). Newcastle disease virus, classified as Avian orthoavulavirus (AOAV-1; formerly avian paramyxovirus type 1 [APMV-1]), is divided into class I and II, which are further subdivided into genotypes and subgenotypes (*2*–*4*). Pigeon paramyxovirus type 1 (PPMV-1) refers to class II genotype VI strains that circulate in pigeons and doves (*5*,*6*). The first well-documented human PPMV-1 infections were reported in the late 1990–2000s as fatal pneumonia in immunosuppressed transplant recipients (*7*–*9*). During the past few years, 2 fatal cases of PPMV-1–associated encephalitis were reported in immunodeficient patients (*10*,*11*). We report an autochthonous case of fatal encephalitis caused by PPMV-1 in France.

## The Study

A 69-year-old man was admitted to an emergency department after repeated falls at home. A week earlier, he attended a shamanism workshop in the Ardèche forest (France). Four days before hospitalization, marked asthenia, diarrhea, diplopia, and dizziness developed in the patient, resulting in falls without loss of consciousness. His family reported a cough for the past year and fatigue and weight loss for several months. The patient had never traveled outside metropolitan (mainland) France. 

**Figure 1:**
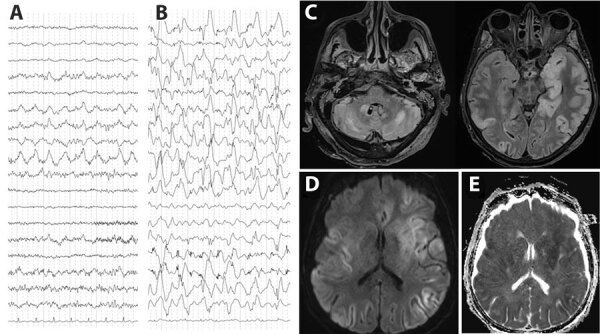
Electroencephalogram (EEG) and brain magnetic resonance imaging (MRI) testing of a patient with encephalitis caused by pigeon paramyxovirus type 1, France. A) The first EEG (day 5 after hospitalization) revealed nonspecific encephalopathy consisting of disorganized theta background rhythm and right temporal rhythmic delta focalization without seizure. B) A later EEG (day 23 after hospitalization) confirmed severe encephalitis with continuous, diffuse, nonreactive, high-amplitude, delta rhythm background of pseudo-periodic delta biphasic and triphasic waves, suggestive of cortical necrosis. C) MRI revealed diffuse bilateral fluid-attenuated inversion recovery hyperintensities, primarily cortical, with subcortical extensions, particularly to the temporal and insular regions, involving the pulvinar nuclei of the thalami, the basal ganglia, the internal capsule, the limbic system, and cerebellum. D, E) Hypersignals in diffusion-weighted images (D) and with reduced apparent diffusion coefficient (E) suggested diffuse cytotoxic brain edema in relation to severe necrotic encephalitis.

At admission, the patient was confused and unable to stand or walk and had a fever of 39°C, right-ear hearing loss, right peripheral facial paralysis, left upper limb ataxia, and multidirectional nystagmus. Deep tendon and plantar reflexes were unremarkable. Routine laboratory tests revealed mild inflammation (hyperleukocytosis at 15 G/L [reference range 4–10 G/L]) and C-reactive protein of 25 mg/L (reference range <5 mg/L). Two cerebrospinal fluid (CSF) analyses revealed no pleocytosis, negative viral and bacterial PCR testing, and negative cultures. We noted isolated hyperproteinorachia of 0.72 g/L (reference range 0.15–0.45 g/L). An electroencephalogram revealed nonspecific encephalopathy (Figure 1, panel A). Brain magnetic resonance imaging revealed no hemorrhage or recent ischemia but showed mild white matter hyperintense lesions at the junction of the mesencephalon and pons on T2 or fluid-attenuated inversion recovery.

Five days after admission, the patient was transferred to the intensive care unit for coma, respiratory failure, and aspiration pneumonia. His neurologic condition deteriorated, and dysarthria, diplopia, deafness, ataxia, swallowing disorders, and quadriparesis developed. He was promptly intubated and mechanically ventilated. Repeat electroencephalogram (Figure 1, panel B) and brain magnetic resonance imaging (Figure 1, panels C–E) confirmed severe encephalitis. A third CSF analysis confirmed hyperproteinorachia of 1.5 g/L and no pleocytosis. Routine laboratory test results were unremarkable, including investigations for rare etiologies of encephalitis (Appendix). Electroneuromyography revealed severe motor and sensory axonal neuropathy of all 4 limbs. A full-body computed tomography scan revealed enlarged centimetric mediastinal and hilar lymphadenopathies and widespread colitis from the sigmoid to the cecum. Despite supportive care and intensive medical management (Appendix), the patient died 26 days after admission from progressively worsening, life-threatening encephalitis and polyradiculoneuropathy of unknown etiology.

To investigate the origin of the encephalitis, we performed metagenomic next-generation sequencing on postmortem midbrain and cervical spinal cord tissues obtained at autopsy, generating 82.5 million midbrain reads and 67.3 million cervical spinal cord reads. We assembled the complete PPMV-1/Human/France/2023 genome from 3,245 reads (0.48%, sequencing depth 28×) from the midbrain and 3,790 reads (0.68%, sequencing depth 34×) from the cervical spinal cord samples. We did not detect any additional pathogens.

**Figure 2:**
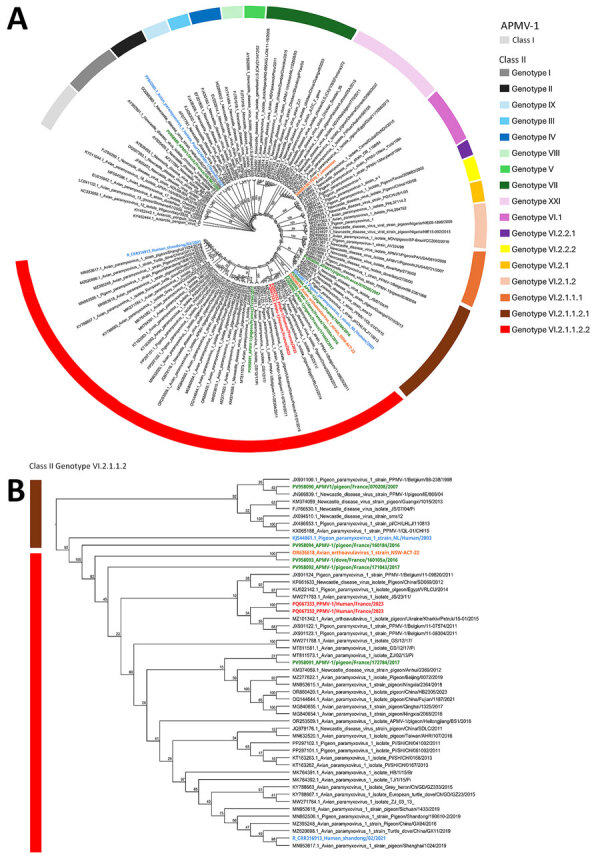
Phylogenetic analysis of fusion gene sequences of PPMV-1from a human case of fatal PPMV-1encephalitis in France and representative APMV-1 strains. A) We conducted phylogenetic analysis on the basis of the nucleic acid sequence of the F gene by using the maximum-likelihood method with the general time reversible with invariable site plus discrete gamma model substitution and the approximate Bayes test for the branch support analysis. Within class II, each genotype is represented by a different color. Names of sequences of interest are colored: red, PPMV-1/Human/France/2023 from the midbrain and cervical cord; green, APMV-1 strains identified in Columbidae species in France n; blue, known APMV-1 strains that caused fatal pneumonia in humans; orange, 2 strains of APMV-1 that caused encephalitis in humans. B) Class II genotype VI.2.1.1.2 PPMV-1/Human/France/2023 isolate from the patient and close reference sequences. The polybasic cleavage motif ^112^RRQKRF^117^ associated with virulent strains is exhibited by all APMV-1 class II, genotype VI, subgenotype 2.1.1.2.2 strains represented in the tree. Represented as a cladogram. Branch node values are branch support as calculated by the approximate Bayes test. GenBank accession numbers are provided. APMV-1, avian paramyxovirus type 1; PPMV-1, pigeon paramyxovirus type 1.

Phylogenetic analyses revealed that PPMV-1/Human/France/2023 belongs to APMV-1 class II, genotype VI, sub-genotype 2.1.1.2.2. The predicted fusion protein contained the polybasic cleavage motif ^112^RRQKRF^117^ associated with virulence in birds (*10**,**12*). PPMV-1/Human/France/2023 was related to PPMV-1 strains from China, Egypt, Belgium, and Ukraine but also APMV-1/pigeon/France/172784/2017 from France (Haute-Garonne department). The sequence from France was obtained through a surveillance program in wild birds: the original sample was collected from a wild pigeon in November 2017, ≈250 km away from Ardèche (Appendix Table 1). PPMV-1/Human/France/2023 is more closely related to Pi/SH/CH/041002/2011 (GenBank accession no. PP297102.1), which was discovered in pigeons from China in 2011 (Figure 2; Appendix Table 2).

**Figure 3:**
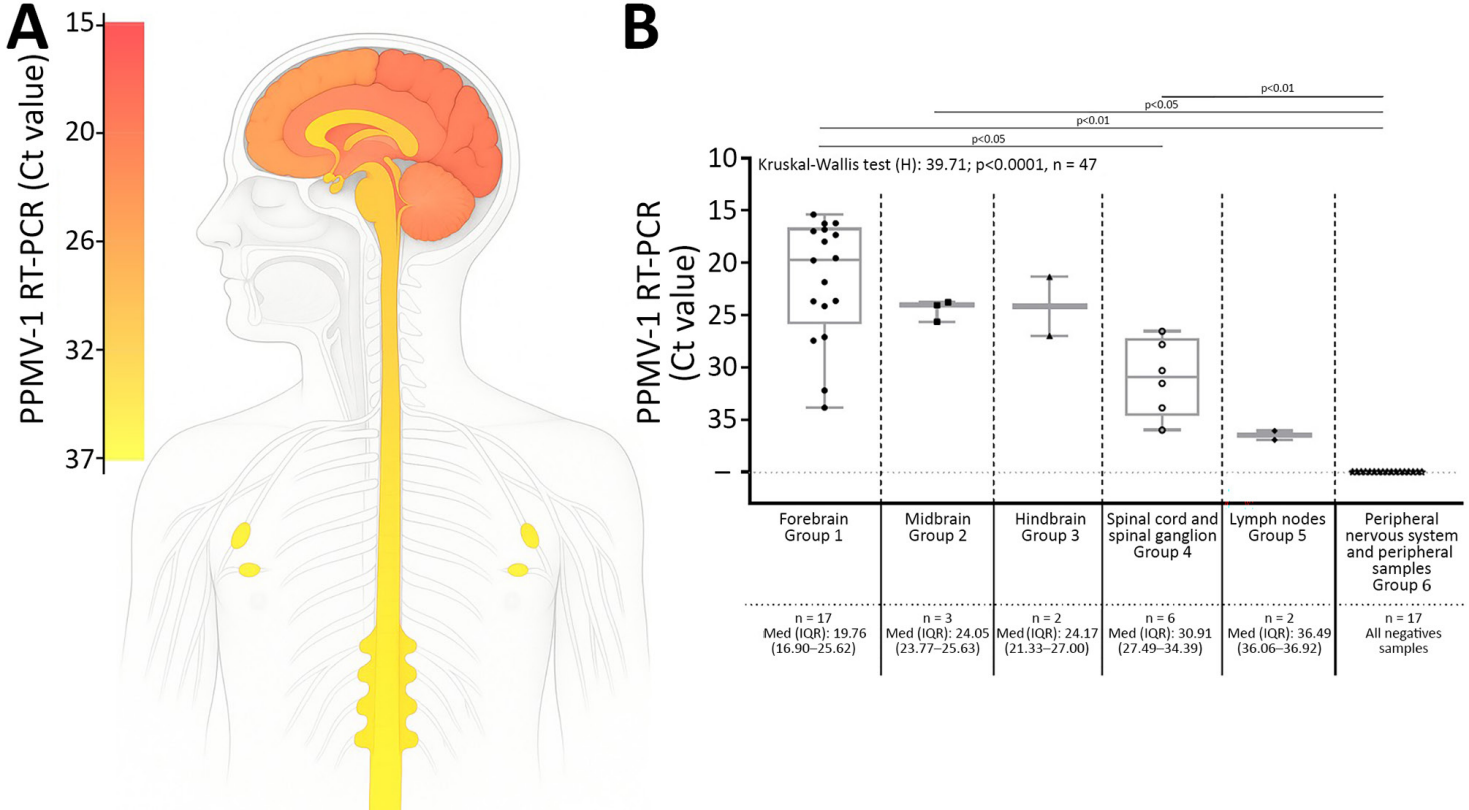
PPMV-1 dissemination in the central and peripheral nervous systems and in axillary lymph nodes in a patient with fatal PPMV-1 encephalitis in France. A) Schematic representation of PPMV-1 viral load in 30 postmortem tissue samples; complete list of all samples is provided (Appendix Table 3, https://wwwnc.cdc.gov/EID/article/32/5/25-1576-App1.pdf). Viral loads were estimated at 5.2 × 10^5^−2.6 × 10^6^ genome copies/gram of tissue in the midbrain and 1.1−5.5 × 10^5^ genome copies/gram of tissue in the cervical spinal cord. B) PPMV-1 viral burden in different anatomic compartments. Samples were classified into 6 groups: group 1, forebrain (including telencephalon and diencephalon); group 2, midbrain; group 3, hindbrain (including metencephalon and myelencephalon); group 4, spinal cord and spinal ganglion; group 5, lymph nodes; group 6, peripheral nervous system and peripheral samples. For the 17 forebrain samples (group 1), the PPMV-1 Ct values were the lowest (median 19.76 [IQR 16.90–25.62]), reflecting the highest viral burden. A Ct value >30 was observed in only 2 forebrain samples, the choroid plexus (Ct = 32.2) and the pituitary gland (Ct = 33.85). The 3 midbrain samples (group 2) and the 2 hindbrain samples (group 3) had higher Ct values: medians 24.05 (IQR 23.77–25.63) and 24.17 (IQR 21.33–27.00). Ct value further increased in the subsequent groups: medians 30.91 (IQR 27.49–34.39) in the 4 spinal cord and 2 spinal ganglion samples (group 4) and 36.49 (IQR 36.06–36.92) in the 2 axillary lymph node samples (group 5), indicating a progressive decrease in viral burden. The last group, consisting of 17 samples (group 6) including peripheral nervous system samples and 5 peripheral samples were all negative. We conducted a nonparametric Kruskal-Wallis test that indicated differences among groups (p<0.0001). Posthoc tests revealed an increasing trend in PPMV-1 Ct value from the brain to the peripheral samples. Pairwise Wilcoxon tests (2-tailed) for differences in means between groups adjusted with Bonferroni correction are displayed. Nonsignificant p values (>0.05) are not shown. Horizontal line within boxes indicate medians, box tops and bottoms represent IQRs, and whiskers indicate minimum and maximum values. Ct, cycle threshold; IQR, interquartile range; PPMV-1, pigeon paramyxovirus type 1.

To investigate the dissemination of PPMV-1 in the patient, we conducted semiquantitative real-time PCR on all clinical samples (Appendix Table 3). PPMV-1 was detected in the CNS, thoracic spinal ganglia, and axillary lymph nodes. We identified the highest viral burdens in the brain, specifically localized to the forebrain. Posthoc tests revealed an increasing trend in PPMV-1 cycle threshold values from the brain to the peripheral samples (Figure 3).

**Figure 4:**
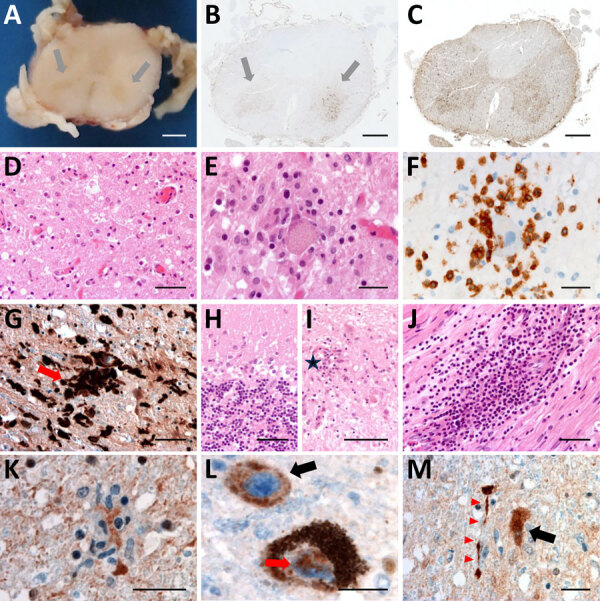
Histologic examination of the nervous tissue in fatal human encephalitis case caused by pigeon paramyxovirus type 1, France. A) Brown discoloration of the anterior horns (gray arrows) of the spinal cord at macroscopic examination. We evaluated the tissue microscopically by using hematoxylin and eosin staining, which highlighted a diffuse inflammatory infiltrate consisting of activated macrophages and microglia in the anterior horns of the spinal cord (throughout its entire length), the brainstem (including medulla oblongata, pons and midbrain), the thalamus, and the cerebellum. Scale bar = 2 mm. B) Anti-CD163 staining revealed marked infiltration of the anterior horns of the spinal cord by activated macrophages (gray arrows). Scale bar = 1.5 mm. C) Anti-Iba1 staining revealed activated microglia involving the anterior horns and the lateral cords of the spinal cord. This inflammatory infiltrate was associated with severe neuronal loss, particularly in the anterior horns of the spinal cord and the dentate nucleus and the Purkinje cell layer of the cerebellum. Scale bar = 1.5 mm. D, E) H&E staining revealed massive loss of motor neurons of the anterior horn (D; scale bar = 50 µm) associated with foci of neuronophagia (E; scale bar = 25 µm). F) Anti-CD3 staining revealed microglial activation was associated with T-cytotoxic lymphocyte (CD3+/CD8+) infiltration of the brain parenchyma in the most severely affected regions, the T lymphocytes surrounding a motor neuron in the gray matter of the spinal cord. Scale bar = 25 µm. G) Anti-Iba1 staining revealed multiple foci of neuronophagia and microglial nodules were observed, indicating high activity of the cellular immune response involving mainly neurons as seen in microglial nodule (red arrow) in the substantia nigra. Scale bar = 50 µm. H) H&E staining revealed that this inflammatory infiltrate was associated with severe neuronal loss, particularly in the Purkinje cell layer of the cerebellum. Scale bar = 50 µm. I) H&E staining revealed massive loss of Purkinje cells associated with Bergmann astrogliosis in the cerebellar cortex and in the anterior horns of the spinal cord and the dentate nucleus, loss of neurons in the dentate nucleus of the cerebellum associated with the presence of microglial nodules (star). Scale bar = 50 µm. J) H&E staining revealed a subtle lymphocytic infiltrate surrounding some of the vessels and a perivascular lymphocytic inflammatory infiltrate of the endoneurium of peripheral nerves. Scale bar = 50 µm. K–M) Anti-NVD staining revealed positivity in a microglial nodule in the anterior horn of the spinal cord (K), in the soma (black arrow) and in the nucleus (red arrow) of neurons in the *substantia* nigra (L), and (in the soma of a neuron (black arrow) and in neurites (red arrowheads) in the dentate nucleus of the cerebellum M). Scale bars = 25 µm). No cytopathic effects were noted. Immunohistochemistry testing with for herpes simplex virus 1, cytomegalovirus, polyomavirus, papovavirus, and rabies virus were negative. H&E, hematoxylin and eosin.

Postmortem examination of the CNS revealed brain edema, friable midbrain tissue, and gray matter discoloration of the spinal cord (Appendix). Histopathologic testing (Appendix Table 4) revealed a diffuse inflammatory infiltrate of activated macrophages, microglia, and CD3+ and CD8+ T lymphocytes, with severe neuronal loss in the spinal cord, brainstem, and cerebellum. Neuronophagia and microglial nodules were frequent. We did not observe cytopathic effect. The choroid plexus and ependymal cells appeared unremarkable. We found perivascular inflammatory infiltrates in peripheral nerves (Figure 4). Newcastle disease virus immunostaining was positive in the spinal cord, midbrain, cerebellum, hypothalamus, and lymph nodes (Figure 4).

## Conclusions

We report a case of fatal encephalitis associated with PPMV-1 in France that belonged to virulent class II, genotype VI, subgenotype 2.1.1.2.2, with polybasic F cleavage motif RRQKRF, in a patient with no known immunosuppression. That motif is associated with high intracerebral pathogenicity in birds (*13*) and was reported in another fatal encephalitis case (*10*). PPMV-1 belongs to the velogenic pathotype of AOAV-1 (Appendix).

The patient had never traveled outside France, indicating autochthonous infection. Although the source of infection is undetermined, the temporal association with a shamanism workshop in the Ardèche forest suggests environmental exposure to avian pathogens, potentially through contact with avian feces. PPMV-1 is shed orally and cloacally, remains stable in pigeon feces, and can spread by windborne dust, extending risk beyond localized environments (*8*).

Two previously reported human APMV-1 encephalitis cases occurred in severely immunosuppressed patients, suggesting that immune status might modulate APMV-1 neuropathogenicity (*10*,*11*). However, the patient we report had no known underlying disease or immunosuppressive treatment. His family reported a deterioration in his general condition, including severe weight loss, in the year before death, without medical evaluation. No laboratory markers of immunosuppression were found. We quantified blood torque teno virus loads to further assess immunosuppression, but results were inconclusive regarding immune status at the time of PPMV-1 infection (*14*). Detecting unusual pathogens in seemingly immunocompetent patients should prompt immune status investigation.

Real-time PCR of postmortem and peripheral samples exhibited strong neurotropism of PPMV-1 without meningitis or systemic infection at the time of neurologic manifestations. PPMV-1 was not detected in the CSF by PCR or metagenomics. PCR of bronchoalveolar lavage was negative 10 days after symptom onset, suggesting weak and transient respiratory replication despite the respiratory tract being the usual entry route. Negative blood PCR results on days 16 and 24 might reflect a brief early viremia.

The patient’s clinical manifestations included severe polyradiculoneuropathy with inflammatory infiltrates in the spinal cord and peripheral nerves, which were not described previously (*10*,*11*). The patient also exhibited enlarged lymphadenopathies, and PPMV-1 was detected at low burden in the right and left axillary lymph nodes; immunostaining confirmed viral tropism in those nodes. Lymph node aspiration could provide a minimally invasive alternative to brain biopsy for diagnosing PPMV-1 encephalitis.

In summary, in the case we report and 2 other reported encephalitis cases, class II APMV-1 with a virulence-associated F-protein cleavage site was identified. APMV-1 infection might be underdiagnosed and should be considered in neurologic disease of unknown etiology, particularly among patients who report exposure to Columbidae fauna.

Appendix Additional information about One Health investigation into fatal encephalitis caused by pigeon paramyxovirus type 1, France.
