# Supplementary material for: One Health Investigation into Fatal Encephalitis Caused by Pigeon Paramyxovirus Type 1, France
Source: Emerg Infect Dis. 2026 May;32(5):753–8. doi: 10.3201/eid3205.251576 (PMC13175106; doi:10.3201/eid3205.251576)
Supplement: Appendix — Additional information about One Health investigation into fatal encephalitis caused by pigeon paramyxovirus type 1, France. [file 25-1576-Techapp-s1.pdf]

Article DOI: https://doi.org/10.3201/eid3205.251576

EID cannot ensure accessibility for supplementary materials supplied by authors. Readers who have difficulty accessing supplementary content should contact the authors for assistance.

One Health Investigation into Fatal Encephalitis caused by Pigeon Paramyxovirus Type 1, France

Appendix

Appendix background

APMV-1 virulent strains and pathobiology

According to international animal health standards, APMV-1 are divided between virulent and avirulent strains based on biologic and genomic criteria (*1*). Canonical pathotypes of the disease have been defined following experimental inoculation in chickens. Asymptomatic enteric and lentogenic strains cause gastrointestinal or respiratory infections without associated disease in birds, respectively: these avirulent strains are commonly used as live vaccines against Newcastle disease in poultry. Mesogenic isolates cause systemic infections with intermediate virulence resulting in non-fatal respiratory diseases, although neurologic disease and death may be observed in young birds. The virulent velogenic strains including PPMV-1 may be either viscerotropic infecting the gastrointestinal mucosa and resulting in fatal hemorrhagic injuries, or neurotropic causing an initial respiratory followed by a secondary neurologic disease (*2–5*). Moreover the polybasic cleavage motif RRQKRF in the fusion protein (that mediates the fusion of the viral envelope with the external cell membrane) is associated with high intracerebral pathogenicity in birds (*6*). The pathobiology of APMV-1 has been characterized on clinically and experimentally infected birds revealing that variations depend mostly on the strain of the infecting virus, and then on the host species, age, immune status, stress, environmental conditions, and the route of transmission (*2*,*3*,*6*).

Appendix Laboratory and Clinical Data

Appendix Laboratory Data

Routine microbiology tests were negative including blood, urine and CSF culture, Meningitis/Encephalitis panel PCR (Biofire Filmarray, Biomérieux) and specific PCRs for EBV and CMV. Investigation of rare etiologies of encephalitis such as PCR for *Tropheryma whipplei* in CSF and saliva, and *Lyssavirus* PCR were negative in blood, CSF and saliva. Serologies for *Lyssavirus*, Lyme disease, and HIV were negative, and syphilis serology indicated a past exposure. Candida antigen and β-D-glucan were positive in the serum while PCRs for *Aspergillus fumigatus*, *Lichtheimia*, *Rhizomucor*, *Rhizopus*, and *Mucor* were negative. Detection of 14.3.3 protein in CSF was negative. Metagenomic next-generation sequencing (mNGS) performed on blood and CSF was negative. Lymphocyte immunophenotyping was normal and no oligoclonal bands was detected in the CSF. Tests for autoimmune causes of encephalitis were all negative including panels for scleroderma, myositis, DOT-myositis, anti-nuclear antibodies, ANCA, anti-ribosome, anti-myelin, anti-MAG, anti-LGI1, anti-CAPR2, and anti-ganglioside GD1b and GD2 antibodies. Serum folate, B1, B6, and B12 vitamins were normal. Blood and urine toxicological screenings, including heavy metals were negative.

Appendix Clinical Data

During hospitalization, the patient received supportive care, anticonvulsants (levetiracetam and lacosamide), multiple vitamin supplementation, intravenous immunoglobulin therapy (0.4 g/kg daily for 5 days), plasmapheresis (four sessions), methylprednisolone (1g boluses during 3 days followed by 1mg/kg daily), empiric large-broad spectrum antibiotic and antifungal treatments (piperacillin-tazobactam, doxycycline, and caspofungin).

Appendix Neuropathological Data

The diffuse inflammatory infiltrate involved the anterior horns of the spinal cord throughout its entire length, the brainstem (including medulla oblongata, pons and midbrain), the thalamus and the cerebellum. In the present case, cortical involvement was mild despite being more prominent in the previously reported biopsy cases (*7*,*8*). A mild perivascular lymphocytic infiltrate was present around some blood vessels without associated endothelial hyperplasia or

fibrinoid necrosis. No demyelination was observed although previous reports have described this feature in pigeons and in rare cases of CNS infection by avian influenza A H5N1 virus in humans (*9–11*). In the peripheral nerves, the inflammatory infiltrates were perivascular and were observed in the endoneurium and perineurium. Immunohistochemistry with anti-HSV1, CMV, Polyomavirus, Papovavirus, and anti-rabies antibodies were negative (Appendix Table 4).

Appendix Tables

Appendix Table 1. French avian APMV-1 full genome sequences – sampling information

Sequence	Class / genotype	Species	Bird category	Sampling date	Sampling location - department (eurostat nuts 3 level identification)	Collected sample
APMV-1/duck/France/06788/2006	II / II	Mallard (*Anas platyrhynchos*)	wild bird	August 2006	Loire-Atlantique	cloacal swab
APMV-1/pigeon/070208/2007	II / VI	Pigeon*	wild bird	July 2007	Dordogne	brain
APMV-1/pigeon/160184/2016	II / VI	Pigeon*	wild bird	February 2016	Lot	brain
APMV-1/dove/France/160105a/2016	II / VI	Dove*	wild bird	January 2016	Rhône	brain
APMV-1/pigeon/171043/2017	II / VI	Pigeon*	poultry	January 2017	Pas-de-Calais	brain
APMV-1/pigeon/172784/2017	II / VI	Pigeon*	wild bird	November 2017	Haute-Garonne	brain

*Partially identified bird species

Appendix Table 2. Percentage identity matrix on the complete genome sequences of PPMV-1

Strains	PQ067332_PPMV-1/Human/France/2023	PQ067333_PPMV-1/Human/France/2023	PP297102.1_Pi/SH/CH/041002/2011	PV958092_APMV-1/pigeon/France/171043/2017	PV958091_APMV-1/pigeon/France/172784/2017	PV958093_APMV-1/dove/France/160105a/2016	PV958094_APMV-1/pigeon/France/160184/2016	PV958090_APMV1/pigeon/France/070208/2007	PV958089_APMV1/duck/France/06788/2006
PQ067332_PPMV-1/Human/France/2023	100	99,94	97,46	96,64	96,41	95,83	94,98	93,69	83,45
PQ067333_PPMV-1/Human/France/2023		100	97,5	96,67	96,44	95,86	95,01	93,73	83,49
PP297102.1_Pi/SH/CH/041002/2011			100	98,42	98,34	97,54	96,39	95,24	83,89
PV958092_APMV-1/pigeon/France/171043/2017				100	97,28	96,84	95,85	94,51	83,62
PV958091_APMV-1/pigeon/France/172784/2017					100	96,41	95,47	94,29	83,57
PV958093_APMV-1/dove/France/160105a/2016						100	95,6	94,43	83,49
PV958094_APMV-1/pigeon/France/160184/2016							100	94,43	83,44
PV958090_APMV1/pigeon/France/070208/2007								100	84,28
PV958089_APMV1/duck/France/06788/2006									100

The pairwise comparison of the complete genome sequences of PPMV-1/Human/France/2023 to PPMV-1 genomes from Columbidae in France and PPMV-1 isolate Pi/SH/CH/041002/2011 detected in Pigeon from People's Republic of China. The table provides the percentage of nucleotide identity for each pair comparisons as obtained using Clustal Omega (*12*). PPMV-1/Human/France/2023 full-length genome sequences assembled independently from the midbrain and cervical spinal cord presented 99.94% nucleotide identity. PPMV-1/Human/France/2023 genome presented a maximum of 97.46% nucleotide identity to PPMV-1 isolate Pi/SH/CH/041002/2011 (PP297102.1) detected in Pigeon from People's Republic of China in 2011. The most closely related strain from France (PV958091_APMV-1/pigeon/France/172784/2017 collected in the Haute-Garonne department in 2017) displayed a 96.64% nucleotide identity to PPMV-1/Human/France/2023. Summary sampling details for the complete French avian APMV-1 genome sequences is avilable in Appendix Table 1.

Appendix Table 3. Collection of clinical samples and PPMV-1 RT-PCR result

Anatomic compartments groups	Clinical samples	PPMV-1 RT-PCR (CT value)
**Post-mortem tissues**		
**Group 1:** *Diencephalon*	Thalamus*	16.97
**Group 1:** *Diencephalon*	Hypothalamus*	23.63
**Group 1:** *Diencephalon*	Pituitary gland*	33.85
**Group 1:** *Telencephalon*	Temporal lobe*	15.38
**Group 1:** *Telencephalon*	Occipital cortex*	16.21
**Group 1:** *Telencephalon*	Caudate nucleus*	16.25
**Group 1:** *Telencephalon*	Lenticular nucleus*	16.82
**Group 1:** *Telencephalon*	Temporal cortex*	17.33
**Group 1:** *Telencephalon*	Cingulate gyrus*	17.96
**Group 1:** *Telencephalon*	Parietal cortex*	19.56
**Group 1:** *Telencephalon*	Hippocampus*	19.76
**Group 1:** *Telencephalon*	Amygdaloid nucleus*	21.84
**Group 1:** *Telencephalon*	Frontal cortex*	23.68
**Group 1:** *Telencephalon*	Superior-internal frontal cortex*	24.13
**Group 1:** *Telencephalon*	Corpus callosum*	27.1
**Group 1:** *Telencephalon*	Frontal white matter*	27.43
**Group 1:** *Telencephalon*	Choroid plexus*	32.2
**Group 2:** *Mesencephalon*	Midbrain* ***(sample 1)***	23.77
**Group 2:** *Mesencephalon*	Midbrain (substantia nigra)*	24.05
**Group 2:** *Metencephalon*	Pons*	25.63
**Group 3:** *Metencephalon*	Cerebellum (dentate nucleus)*	21.33
**Group 3:** *Myelencephalon*	Medulla oblongata*	27
**Group 4:** *Spinal cord*	Cervical spinal cord* ***(sample 2)***	26.54
**Group 4:** *Spinal cord*	Cervical spinal cord*	27.81
**Group 4:** *Spinal cord*	Lumbar spinal cord*	30.3
**Group 4:** *Spinal cord*	Thoracic spinal cord*	31.51
**Group 4:** *Spinal ganglion*	Lower thoracic spinal ganglion*	33.86
**Group 4:** *Spinal ganglion*	Upper thoracic spinal ganglion*	35.98
**Group 5:** *Lymph nodes*	Right axillary lymph node*	36.06
**Group 5:** *Lymph nodes*	Left axillary lymph node*	36.92
**Group 6:** *Peripheral Nervous System*	Right ulnar nerve*	Negative
**Group 6:** *Peripheral Nervous System*	Left ulnar nerve*	Negative
**Group 6:** *Peripheral Nervous System*	Right axillary nerve*	Negative
**Group 6:** *Peripheral Nervous System*	Left axillary nerve*	Negative
**Group 6:** *Peripheral Nervous System*	Right radial nerve*	Negative
**Group 6:** *Peripheral Nervous System*	Left radial nerve*	Negative
**Group 6:** *Peripheral Nervous System*	Right median nerve*	Negative
**Group 6:** *Peripheral Nervous System*	Left median nerve*	Negative
**Group 6:** *Peripheral Nervous System*	Right sural nerve (leg)*	Negative
**Group 6:** *Peripheral Nervous System*	Left sural nerve (leg)*	Negative
**Group 6:** *Peripheral Nervous System*	Right lower sciatic / popliteal nerve*	Negative
**Group 6:** *Peripheral Nervous System*	Left lower sciatic / popliteal nerve*	Negative
**Peripheral and clinical samples collected during hospitalization**		
**Group 6:** *Peripheral samples*	Sigmoid biopsy†	Negative
**Group 6:** *Peripheral samples*	Bronchoalveolar lavage fluid†	Negative
**Group 6:** *Peripheral samples*	Cerebrospinal fluid†	Negative
**Group 6:** *Peripheral samples*	Plasma†	Negative
**Group 6:** *Peripheral samples*	Whole blood†	Negative

*Post-mortem tissues frozen at −80°C
†Peripheral and clinical samples collected during hospitalization frozen at −20°C
CT: Cycle threshold value
Peripheral and clinical samples collected during hospitalization. During the 26 d of hospitalization, several samples were collected: a bronchoalveolar lavage fluid (BAL), a sigmoid biopsy, cerebrospinal fluid (CSF), plasma and whole blood.
Post-mortem tissues. An autopsy was performed in agreement with the current French laws, after obtaining informed consent from the patient's next of kins and querying the National Refusal Register (Agence de la Biomédecine). Each sample was frozen at −80°C for virological analysis and fixed in formalin for neuropathological analysis.
All clinical samples on which semiquantitative PPMV-1 RT-PCR was performed are included. There are 42 post-mortem tissue samples from the autopsy and 5 samples collected from the patient during their hospitalization.
The 47 samples were classified into 6 anatomic compartment groups. defined as follows:
Group 1: Forebrain (Telencephalon, diencephalon)
Group 2: Midbrain
Group 3: Hindbrain (Metencephalon, Myelencephalon)
Group 4: Spinal cord and spinal ganglion
Group 5: Lymph nodes
Group 6: Peripheral nervous system and peripheral samples

Appendix Table 4. Immunohistochemistry antibodies

Antibody	Clone	Clonality	Epitope	Source	Pretreatment	Dilution	Incubation time
**CD3**	2GV6	Monoclonal (mouse)	Epsilon chain of the human CD3	Ventana	CC1® 30 min	prediluted	32 min
**CD4**	4B12	Monoclonal (mouse)	T helper cells	Dako	CC1® 36 min	1:40	60 min
**CD8**	C8/144B	Monoclonal (mouse)	Suppressor/cytotoxic T cells	Dako	CC1® 36 min	1:50	32 min
**CD20**	L26	Monoclonal (mouse)	Transmembrane proteins expressed on B cells	Dako	CC1® 36 min	1:100	32 min
**CD68**	KP1	Monoclonal (mouse)	NA	Dako	CC1® 36 min	1:1000	20 min
**CD163**	MRQ-26	Monoclonal (mouse)	NA	Cell Marque	CC1® 64 min	Ready-to-use	32 min
**CMV**	CCH2+ DDG9	Monoclonal (mouse)	CMV immediate early antigen and early antigen	Dako	Protease 4 min	1:50	32 min
**HSV1**	1OA3	Monoclonal (mouse)	ICP8	Roche	CC1® 36 min	Ready-to-use	32 min
**Measles virus**	EP207	Monoclonal (rabbit, IgG1)	NA	Quartett	CC1® 36 min	1:50	60 min
**NDV (APMV-1)**	6H12	Monoclonal (mouse)	Hybridization of Sp2/0 myeloma cells with spleen cells of Balb/c mice immunized with Newcastle disease virus (La- sota strain)	Novus Biologic	CC2® 44 min	1:100	60 min
**Papova virus (SV40)**	MRQ-4	Monoclonal (mouse)	SV40 large T antigen	Cell Marque	CC1® 76 min	Ready-to-use	2 h
**Rabies virus**	Rab50	-	NA	Bio-Rad	CC2® 44 min	1:100	1 h 30 min
**Toxoplasma gondii**	NA	Polyclonal (rabbit)	NA	Biogenex	CC1® 8 min	Ready-to-use	32 min
**VZV**	7 clones cocktail	Monoclonal (mouse)	NA	Monosan	-	Ready-to-use	32 min

CMV: Cytomegalovirus; **HSV1:** Herpes-simplex virus 1; **NDV:** Newcastle disease virus; **VZV:** varicella-zoster virus
NA, not available.

Appendix methods

Metagenomic next-generation sequencing (mNGS)

Total RNA was extracted from post-mortems brain tissue, the midbrain and cervical spinal cord, using the ELITe InGenius instrument (ELITechGroup) after an initial step of tissue disruption with Bertin beads and a Precellys homogenizer (Bertin Technologies). A DNase treatment was applied with TURBO DNase (Invitrogen). NGS libraries were constructed with the SMARTer Stranded Total RNA-Seq Kit - Pico Input Mammalian Kit v.3 (Takara Bio) and sequenced in 1x150bp on a NextSeq500 instrument (Illumina) using a High Output flow cell. The search for pathogen was performed using Microseek bioinformatic pipeline (*13*,*14*). The mNGS performed on midbrain and cervical spinal cord post-mortem tissues from the autopsy generated 82.5 and 67.3 raw millions reads, respectively.

PPMV-1 genome assembly and annotation

The full genome of PPMV-1 was reconstructed from the output of Microseek, with final manual check. Coding regions were annotated by homology with PPMV-1 reference genomes. Consistency and absence of stop codon were checked. The annotated genomes of PPMV-1/Human/France/2023 were deposited to Genbank under accession numbers PQ067332 (midbrain) and PQ067333 (cervical spinal cord).

APMV-1 detection and identification in poultry and wild birds, France

Since 2006, comprehensive characterization by sequencing is performed for APMV-1 viruses identified through animal disease surveillance in birds in France. According to European Union regulations, surveillance of Newcastle disease in poultry is based on a compulsory event-based passive surveillance scheme. Although no regulation requires it, surveillance of mortality in wild birds may also incidentally include the diagnosis of APMV-1 infections. Tracheal and cloacal swabs, along with organs such as the brain, lungs and intestines, have been collected for each suspected case of Newcastle disease in poultry or of APMV-1 infection in wild birds. The swabs were resuspended in an antibiotic-supplemented isotonic phosphate buffered saline solution or cell culture medium and the organs were crushed and filtered using the same solution. These samples were then inoculated into the allantoic cavity of 9–11-day-old specific-pathogen-free or specific antibody negative embryonated chicken eggs, to isolate the virus, using a standard procedure (WOAH, 2021). Hemagglutinating allantoic fluids were tested using a haemagglutination inhibition assay with a reference panel of 35 hyperimmune sera against different subtypes of avian influenza viruses and different types of avian paramyxoviruses (including APMV-1) in accordance with international standards (WOAH, 2021). Viral RNA was extracted from APMV-1-positive allantoic fluid using the RNeasy Mini Kit (Qiagen). RT-PCR specific for the F gene, including the sequence surrounding the cleavage site of F protein, was performed following an internal procedure of the National Reference Laboratory (available upon request). The amplicon was purified after electrophoresis using the QIAquick gel extraction kit, following the manufacturer's instructions (Qiagen). The purified amplicons were sequenced using a BigDye Terminator v3.1 kit (Applied Biosystems, USA) with the same primers as the RT-PCR, and results were analyzed using a 3500 Series Genetic Analyzer. All nucleotide sequences obtained surrounding the cleavage site of the F gene of APMV-1 from birds were compared with the nucleotide sequence from the human case, to select the closest sequences for

full genome sequencing. The full genome sequences of the five selected avian viruses were determined using Illumina Stranded Total RNA Prep and NextSeq 2000. The sequences were deposited in GenBank under accession numbers PV958059 to PV958094.

Phylogenetic analyses

The nucleotide sequences of the F gene of PPMV-1/Human/France/2023 were aligned using MAFFTv7 (*15*) to PPMV-1 sequences obtained from wild *Columbidae* from France and additional PPMV-1 sequences from GenBank, including human clinical strains from several geographic locations. Phylogenetic analysis based on the F gene sequences was performed using geneious prime software (version 2025.2), the Maximum-Likelihood method with the GTR (+G+I) model substitution and the approximate Bayes test for the branch support analysis (*16*). The phylogenetic tree was annotated with the geneious prime software (version 2025.2). The genotypes were designated according to newly proposed classification criteria (*17*).

PPMV-1 semiquantitative RT-PCR

Total nucleic acids were extracted from post-mortem tissues, sigmoid biopsy, plasma, whole blood, BAL, and CSF using the ELITe InGenius instrument (ELITechGroup).

PCR reactions were performed in a final volume of 50 μL, containing 1 μL of SuperScript III RT/Platinum Taq Mix, 25 μL of 2X Reaction Mix (a buffer containing 0.4 mM of each dNTP, 6 mM MgSO4, and stabilizers), 1 μL of each forward and reverse primer at 10 μM, and 0.5 μL of probe at 10 μM. The remaining volume was adjusted with molecular-grade water.

Semiquantitative TaqMan RT-PCR was performed using the SuperScript III Platinium One-Step qRT-PCR kit (Thermo Fisher Scientific) with 40 cycles of amplification on the LightCycler® 480 (Roche diagnostics). The RT-PCR program consisted of a reverse transcription step at 50°C for 15 minutes and an initial denaturation at 95°C for 2 minutes, followed by 40 cycles of amplification: denaturation at 95°C for 15 seconds and annealing/extension at 57°C for 60 seconds.

The primers and probes used for the detection of the PPMV-1 are as follows:

Forward Primer: 5′-TCCACAATGGAGCTAATGAACAT-3′

Reverse Primer: 5′-GAATAGGCGAACCACATCTGA-3′

Probe: 5′ FAM-TGCTATAATAGTGTATGATAACAC-MGB-Eclipse 3′

This RT-PCR targeting a conserved 171bp region in the viral RNA polymerase gene (L gene) of PPMV-1. Primers and probes were designed based on the PPMV-1/Human/France/2023 sequence obtained by mNGS from the human case. The human ribonucleoprotein gene was used as an internal control to validate the extraction and amplification steps. The primers and probes used for the detection of the human ribonucleoprotein gene, which is used as an internal control in our Semiquantitative TaqMan RT-PCR PPMV-1 protocol, are as follows:

Forward Primer: 5′-AGATTTGGACCTGCGAGCG-3′

Reverse Primer: 5′-GAGCGGCTGTCTCCACAAGT-3′

Probe: 5′-FAMTTCTGACCTGAAGGCTCTGCGCG-BHQ1–3′

Negative controls were included in the experiment.

Quantification of PPMV-1 viral load

From the midbrain (sample 1) and the cervical post-mortem tissue samples (sample 2), total RNA was reverse transcribed using the SuperScript IV First-Strand Synthesis System (Thermo Fischer Scientific), using random primers. Quantitative PCR were done in SYBR Green format with 45 cycles of amplification, using primers FWD: 5′-GCCATGACTGCGTATGAGAC-3′ and REV: 5′- CGGACTGCTAGAGAATGTCTGA-3′ targeting a 149bp conserved region in the nucleoprotein gene (*18*). Primers were adapted to the patient’s sequence. Human β-actin was included in the RT-PCR experiments as a positive control of the cDNA. No Template Control (NTC) wells included in the experiment. Positive amplicons after 45 cycles were purified on gel, confirmed by Sanger sequencing, and serial-dilutions were made to generate standard curves for quantification leading to an estimated PCR efficiency of 1.86 with $R^2 = 0.995$. First, quantitative RT-PCR targeting a 149bp conserved region in the nucleoprotein gene resulted in Ct = 23.0 (sample 1) and Ct = 25.6 (sample 2). Assuming a biopsy weight of [0.1–0.5] gram, this translates to viral loads of [$5.2x10^5$ - $2.6x10^6$] and [$1.1x10^5$ - $5.5x10^5$] genome copies per gram of tissue for sample 1 and sample 2, respectively.

Staining and Immunohistochemistry

A hematoxylin and eosin staining was performed on all brain sections and a periodic acid-schiff, Grocott's methenamine silver stain, and Gram staining were performed for the

detection of bacteria and fungi in areas with mixed inflammatory infiltrate. Immunohistochemistry for the detection of *Toxoplasma gondii* and viruses, including *Herpes simplex virus 1* (HSV1), cytomegalovirus (CMV), rabies virus, measles virus, *Varicella zoster virus* (VZV), *Papova virus* as well as an immunostaining with a monoclonal mouse antibody IgG2a to Newcastle disease virus (NDV) was performed in a Ventana BenchMark stainer (Roche) automated station (Appendix Table 4) (*19*). The biotinylated secondary antibody was included in the detection kit (Ventana Medical Systems Basic DAB Detection Kit 250–001). In addition, an immunohistochemistry with the specific cellular markers CD68, CD163 and Iba-1 for macrophages and activated microglia, as well as a CD20, CD3, CD4 and CD8 for lymphocytes was performed to identify the cellular immune response in the tissues (Appendix Table 4).

Quantification of Torque Teno Virus (TTV) viral load

Nucleic acids were extracted by EMAG (Biomérieux, France). Torque teno virus (TTV) DNA load in plasma and whole blood were measured using the TTV R-GENE kit (Biomérieux, France) (*20*). TTV viral loads in plasma and whole blood collected on days 7 and 15 of ICU admission were 7.33 log copies/mL and 8.26 log copies/mL, respectively. Only samples collected after 7 and 15 days of ICU were available. Given that 15% of ICU patients have high TTV blood viral load during the first month of their ICU stay, a low-to-absent TTV in blood samples would have allowed us to conclude that the patient was not immunosuppressed (*21*). Our results showing high TTV load in plasma and whole blood did not allow us to infer the immunological status of the patient at the time of PPMV-1 infection.

Statistical analysis

The 47 clinical and post-mortem samples were classified into six anatomic compartment groups (group 1: Forebrain; group 2: Midbrain; group 3: Hindbrain; group 4: Spinal cord and spinal ganglion; group 5: Lymph nodes and group 6: Peripheral nervous system and peripheral samples). Quantitative data are presented as median (med) and interquartile range (IQR). A Kruskal-Wallis test was used to compare PPMV-1 viral burden (Ct value) between the six groups corresponding to the different anatomic compartments. Wilcoxon-Mann-Whitney tests with Bonferroni correction were used to compare the mean Ct values between anatomic compartments. Statistical significance was defined by a p-value <0.05. All statistical analyses were performed using GraphPad Prism 10.0.3 (GraphPad Prism Software Inc.).

References

1. Swayne DE. Diseases of poultry. 14th edition. Hoboken: Wiley-Blackwell; 2020.

2. Alexander DJ, Bell JG, Alders RG. A technology review: Newcastle disease, with special emphasis on its effect on village chickens. Rome: Food and Agriculture Organization of the United Nations; 2004. p. 63.

3. Saif YM. Diseases of poultry, 12th ed. Ames (Iowa): Blackwell publishing; 2008.

4. Alexander DJ, Senne DA. Newcastle disease, other avian paramyxoviruses, and pneumovirus infection. In: Swayne DE, Boulianne M, Logue CM, McDougald LR, Nair V, Suarez DL, et al. editors. A laboratory manual for the isolation, identification, and characterization of avian pathogens, 5th ed. 2008. p. 135–41.

5. World Organization for Animal Health. Newcastle disease (infection with Newcastle disease virus). In: Manual of diagnostic tests and vaccines for terrestrial animals, 6th ed. 2008. p. 576–89.

6. World Organization for Animal Health. Newcastle disease (infection with Newcastle disease virus). In: Manual of diagnostic tests and vaccines for terrestrial animals, 7th ed. Paris; 2021.

7. Winter S, Lechapt E, Gricourt G, N'debi M, Boddaert N, Moshous D, et al. Fatal encephalitis caused by Newcastle disease virus in a child. Acta Neuropathol. 2021;142:605–8. PubMed https://doi.org/10.1007/s00401-021-02344-w

8. Hurley S, Eden JS, Bingham J, Rodriguez M, Neave MJ, Johnson A, et al. Fatal human neurologic infection caused by pigeon avian paramyxovirus-1, Australia. Emerg Infect Dis. 2023;29:2482–7. PubMed https://doi.org/10.3201/eid2912.230250

9. Yuzbasioglu-Ozturk G, Gurel A. Histopathological and immunohistochemical detection of pigeon paramyxovirus-1 (pPMV-1) in pigeons. 2022 Mar 8 [cited 2026 Jan 24] https://arccjournals.com/journal/indian-journal-of-animal-research/BF-1469

10. Martiny K, Schlachter AD, Jensen TK, Lean FZX, Núñez A, Hammer AS, et al. Two outbreaks of pigeon paramyxovirus 1 with high mortality in captive pigeons (*Columbia livia*) in Denmark, 2022–2023. Transbound Emerg Dis. 2025;2025:5629889. PubMed https://doi.org/10.1155/tbed/5629889

11. To KF, Chan PK, Chan KF, Lee WK, Lam WY, Wong KF, et al. Pathology of fatal human infection associated with avian influenza A H5N1 virus. J Med Virol. 2001;63:242–6. PubMed https://doi.org/10.1002/1096-9071(200103)63:3<242::AID-JMV1007>3.0.CO;2-N

12. Madeira F, Madhusoodanan N, Lee J, Eusebi A, Niewielska A, Tivey ARN, et al. The EMBL-EBI job dispatcher sequence analysis tools framework in 2024. Nucleic Acids Res. 2024;52(W1):W521–5. PubMed https://doi.org/10.1093/nar/gkae241

13. Fourgeaud J, Regnault B, Ok V, Da Rocha N, Sitterlé É, Mekouar M, et al. Performance of clinical metagenomics in France: a prospective observational study. Lancet Microbe. 2024;5:e52–61. PubMed https://doi.org/10.1016/S2666-5247(23)00244-6

14. Pérot P, Bigot T, Temmam S, Regnault B, Eloit M. Microseek: a protein-based metagenomic pipeline for virus diagnostic and discovery. Viruses. 2022;14:1990. PubMed https://doi.org/10.3390/v14091990

15. Katoh K, Rozewicki J, Yamada KD. MAFFT online service: multiple sequence alignment, interactive sequence choice and visualization. Brief Bioinform. 2019;20:1160–6. PubMed https://doi.org/10.1093/bib/bbx108

16. Guindon S, Dufayard JF, Lefort V, Anisimova M, Hordijk W, Gascuel O. New algorithms and methods to estimate maximum-likelihood phylogenies: assessing the performance of PhyML 3.0. Syst Biol. 2010;59:307–21. PubMed https://doi.org/10.1093/sysbio/syq010

17. Dimitrov KM, Abolnik C, Afonso CL, Albina E, Bahl J, Berg M, et al. Updated unified phylogenetic classification system and revised nomenclature for Newcastle disease virus. Infect Genet Evol. 2019;74:103917. PubMed https://doi.org/10.1016/j.meegid.2019.103917

18. Sheng W, Wang K, Gui Y, Qi X, Shen L, Zhang Y, et al. Molecular characteristics and phylogenetic analysis of pigeon paramyxovirus type 1 isolates from pigeon meat farms in Shanghai (2009–2012). Sci Rep. 2024;14:10741. PubMed https://doi.org/10.1038/s41598-024-61235-8

19. Kuiken T, Breitbart M, Beer M, Grund C, Höper D, van den Hoogen B, et al. Zoonotic infection with pigeon paramyxovirus type 1 linked to fatal pneumonia. J Infect Dis. 2018;218:1037–44. PubMed https://doi.org/10.1093/infdis/jiy036

20. Kulifaj D, Durgueil-Lariviere B, Meynier F, Munteanu E, Pichon N, Dubé M, et al. Development of a standardized real time PCR for Torque teno viruses (TTV) viral load detection and quantification: a new tool for immune monitoring. J Clin Virol. 2018;105:118–27. PubMed https://doi.org/10.1016/j.jcv.2018.06.010

21. Mallet F, Diouf L, Meunier B, Perret M, Reynier F, Leissner P, et al. Herpes DNAemia and TTV viraemia in intensive care unit critically ill patients: a single-centre prospective longitudinal study. Front Immunol. 2021;12:698808. PubMed https://doi.org/10.3389/fimmu.2021.698808
